# Intraoperative 5-aminolevulinic acid fluorescence-guided aspirate tissue monitoring in high-grade glioma surgery: The first-in-human study on clinical performance and safety

**DOI:** 10.1093/noajnl/vdag029

**Published:** 2026-02-16

**Authors:** Antti-Pekka Elomaa, Samu Lehtonen, Einar Osland Vik-Mo, Fady T Charbel, Mikael von und zu Fraunberg, Mikko Visuri, Jaakko Luoma, Milla Kelahaara, Ilkka Haapala, Antti Huotarinen, Susanna Rantala, Joonas Konki, Juho Leskinen, Dmitry Semenov, Joonas Haapasalo, Sami Puustinen

**Affiliations:** Department of Neurosurgery, Helsinki University Hospital, and University of Helsinki, Helsinki, Finland; Faculty of Health Sciences, Clinical Medicine, University of Eastern Finland, Kuopio, Finland; Department of Neurosurgery, Oslo University Hospital, Oslo, Norway; Faculty of Medicine, Institute for Clinical Medicine, University of Oslo, Oslo, Norway; Department of Neurosurgery, University of Illinois Chicago, Chicago, IL, USA; Neurosurgery at Neurocenter and University of Oulu, Research Unit of Clinical Medicine, Oulu University Hospital, Oulu, Finland; Department of Neurosurgery, Turku University Hospital, and Clinical Neurosciences, University of Turku, Turku, Finland, Turku, Finland; Department of Neurosurgery, Turku University Hospital, and Clinical Neurosciences, University of Turku, Turku, Finland, Turku, Finland; Faculty of Science, Forestry and Technology, School of Computing, University of Eastern Finland, Joensuu, Finland; Department of Neurosurgery, Tampere University Hospital, Tampere, Finland; Faculty of Medicine and Health Technology, Tampere University, Tampere, Finland; Department of Neurosurgery, Tampere University Hospital, Tampere, Finland; Faculty of Medicine and Health Technology, Tampere University, Tampere, Finland; Faculty of Health Sciences, Clinical Medicine, University of Eastern Finland, Kuopio, Finland; Faculty of Health Sciences, Clinical Medicine, University of Eastern Finland, Kuopio, Finland; Department of Neurosurgery, Kuopio University Hospital, Kuopio, Finland; Faculty of Science, Forestry and Technology, School of Computing, University of Eastern Finland, Joensuu, Finland; Department of Neurosurgery, Turku University Hospital, and Clinical Neurosciences, University of Turku, Turku, Finland, Turku, Finland; Faculty of Science, Forestry and Technology, School of Computing, University of Eastern Finland, Joensuu, Finland; Faculty of Science, Forestry and Technology, School of Computing, University of Eastern Finland, Joensuu, Finland; Department of Neurosurgery, Tampere University Hospital, Tampere, Finland; Faculty of Medicine and Health Technology, Tampere University, Tampere, Finland; Faculty of Health Sciences, Clinical Medicine, University of Eastern Finland, Kuopio, Finland; Department of Neurosurgery, Kuopio University Hospital, Kuopio, Finland

**Keywords:** 5-aminolevulenic acid, aspirate tissue monitoring, fluorescence-guided surgery, glioblastoma, glioma

## Abstract

**Introduction:**

The resection of high-grade gliomas (HGGs) is limited by diffuse tumor growth and the need to avoid the eloquent tracts. Although fluorescence-guided surgery 5-ALA administration can enhance tumor-resection rates, its visual detection capability may be hindered by tissue obstructions, bleeding, and attenuation of fluorescence by ambient light. To address the limitations of visual fluorescence detection, we investigated the usefulness of an aspirate tissue-monitoring (ATM) device, which provides near-real-time auditory feedback on fluorescence from suction waste.

**Materials and Methods:**

Resections of HGGs were recorded, and data were collected using an ATM. Data were collected from 20 patients for performance analysis in an observational study, and an interventional trial validated clinical applicability with 8 patients who underwent resection with the ATM connected to an ultrasonic aspirator. An expert panel defined tissue fluorescence visibility from the videos.

**Results:**

The ATM detected fluorescence 483% more frequently than visual inspection and for 613% longer. In comparison with the visual assessments, the specificity and sensitivity of the ATM for fluorescence were 100% and 83%, respectively. In histopathological assessments, all the fluorescent areas contained HGGs. The expert panel showed good agreement (average overall agreement rate = 96.7%) for the inference that the ATM is a safe technique that adds value to tissue-mapping techniques.

**Discussion and Conclusion:**

This first-in-human clinical trial evaluated the ATM as an adjunct to visual detection of 5-ALA fluorescence. The ATM provided supplementary fluorescence information during surgery, and its performance under while-light illumination was at least as good as visual analysis under blue-light.

Key Points

- Unintended tumor residuals in surgery lead to worse clinical outcomes.
- Fluorescence-guided surgery improves resection rates but is limited by a complex workflow.
- Aspirate tissue monitoring was investigated as an alternative to visual fluorescence analysis.

Importance of the Study

We describe a novel fluorescence-guided aspirate tissue-monitoring (ATM) device that was used for the detection of 5-aminolevulinic acid (5-ALA) fluorescence in high-grade glioma surgery. Visual detection of 5-ALA-induced fluorescence is limited by tissue obstructions and bleeding, and failure to detect fluorescence can result in unintended local residual tumors. Moreover, the presence of intense lights in the operation room conceals fluorescent emissions and necessitates repetitive switching of the lights in the operating room. We investigated the usefulness of the first commercially available ATM device (HIVEN^®^, MARGINUM Ltd, Finland) that provides real-time feedback on 5-ALA-induced fluorescence from suction aspirates, thereby addressing the limitations of visual fluorescence analysis. This manuscript presents our comprehensive clinical laboratory findings and highlights the clinical benefits from a first-in-human clinical trial. We also present results from a comparison of biopsy findings, visual detection of 5-ALA–induced fluorescence using an operating microscope, and feedback from the ATM device.

## Introduction

### Treatment of High-Grade Gliomas

With a global incidence of 160 000 cases per year, gliomas are the most common malignant primary central nervous system tumors.^1^ The incidence of high-grade gliomas (HGGs) increases with age, and the WHO classification includes three categories of adult-type diffuse gliomas: isocitrate dehydrogenase (IDH)-mutant astrocytoma; IDH-mutant, 1p/19q-codeleted oligodendroglioma; and IDH wild-type (wt) glioblastoma, of which IDH-wt grade 4 glioma is the most frequent.^2^ Treatment guidelines of the European Association of Neuro-Oncology (EANO) recommend maximally safe surgery in conjunction with radiation and chemotherapy as the first line of treatment.^3^ The overall survival (OS) in patients with HGG after supramarginal resection is longer than that after subtotal or gross total resection (GTR). Moreover, the synthesis of benefit–risk profiles of surgical strategies by the Response Assessment in Neuro-Oncology (RANO) resect group supports maximally safe resection as the golden standard of care for all grade 2-4 gliomas.^4^ Evidence suggests that increasing the extent of resection (EOR) is associated with improved outcomes in older adults (>75 years) and young patients, although young patients tend to show better results. The clinical outcomes improve progressively when patients undergo at least partial resection instead of biopsy,^5^ GTR instead of partial total resection,^6^ and supramarginal total resection instead of GTR, with the average progression-free survival (PFS) increasing from 10 to 25 months and the OS increasing from 13 to 29 months in selected series.^7^ A neurologically uneventful EOR beyond the CT or MRI contrast-enhancing region and covering at least 40% of the fluid-attenuated inversion recovery (FLAIR)/T2 hyperintense zone has shown favorable prognostic value for grade 2-3 gliomas with potentially aggressive features.^4^^,^^8^ Supramarginal resection appears to yield the best survival outcomes and is being investigated in comparison with historical institutional resection strategies in a randomized clinical trial (NIH ClinicalTrials.gov: NCT04243005).

To achieve targeted supramarginal resection, contemporary evidence advocates the need for advanced tissue-detection methods such as intraoperative magnetic imaging (ioMRI), intraoperative monitoring (IOM), and fluorescence guidance.^7^ Despite using these adjunct techniques, up to 60% of HGG resections require one or more reoperations^9^; however, the rate of reoperations for grade 2-4 gliomas shows significant institutional differences. For example, in some clinical series, the revision rate was much lower at 10%,^10^ whereas in questionnaires, late revisions for grade 4 tumor residuals are considered feasible for up to 16% of patients.^11^ However, despite revisions, unintended residual tumors continue to be a challenge in surgical treatment of HGGs.

### Fluorescence-Guided Surgery with 5-Aminolevulinic Acid for HGGs

Fluorescence-guided surgery (FGS) utilizes fluorescence-inducing drugs such as 5-aminolevulinic acid (5-ALA) to highlight tissue features during operations. After ingestion of 5-ALA, the tumor accumulates protoporphyrin IX (PpIX), which absorbs blue light and emits reddish fluorescent light. By monitoring PpIX fluorescence, active HGG cells can be visualized under blue light with a modern surgical microscope.^12^ PpIX produces a characteristic pink glow in tumor cells, and identification of PpIX fluorescence remains the most spatially precise near-real-time method for detecting active tumor areas during HGG surgery. The use of 5-ALA in HGG surgery has been approved by both the European Medical Agency (EMA) and the U.S. Food and Drug Administration (FDA), and its use has led to increased tumor-resection rates and better progression and OS in randomized control trials.^13–15^

5-ALA consumption and the accumulation of fluorescent PpIX is usually observed in tumor cells and not healthy parenchyma. The same phenomenon has been observed in various central nervous system (CNS) tumors, all of which show significantly higher ranges of fluorophore levels in comparison with healthy tissues.^16^ However, information based on PpIX fluorescence alone is not entirely conclusive for distinguishing between functional and non-functional areas.^17^ Thus, the use of fluorescence may result in the removal of healthy tissues, which can potentially lead to morbidity directly through resection of functional tissues or indirectly by injuring critical vessels. These risks can be minimized by mandatory surgeon education based on the 5-ALA drug guidelines and further reduced with supplementary techniques for detecting fluorescence.

### Limitations of Visual Analysis of 5-ALA Fluorescence

**Figure 1.**
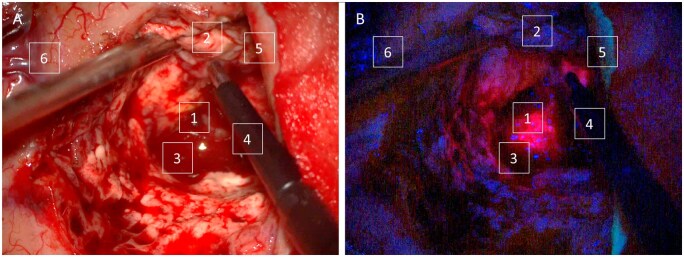
Visual detection of PpIX fluorescence can be limited by various intraoperative factors. Fluorescence can help distinguish the tumor (1) from non-fluorescent brain matter (2), but visual analysis of this fluorescence requires switching from white- (A) to blue-light illumination (B). The fluorescence is visually obstructed by blood (3), instruments (4), tissue ledges, and hemostats (5). Under blue light, blood vessels (6) are harder to detect, and damage to these critical structures can lead to ischemic complications and new neurological deficits.

5-ALA fluorescence is used to detect areas of tumor margins beyond the obvious regions, such as the necrotic core. Blue light is required to detect PpIX fluorescence, and although visual detection of PpIX fluorescence by experts shows high specificity, the sensitivity of this approach for detecting obstructed or weaker fluorescence is poor.^18^ At tumor margins, up to 50% of tumor-related fluorescence can remain undetected in visual assessments with a contemporary surgical microscope.^19^^,^^20^ The microscope’s white-light imaging mode allows detection of blood and debris that obstruct fluorescence excitation and is preferred for maintaining situational awareness of anatomy and critical structures, such as passing (*en passage*) vessels. Injuries to vessels can cause peritumoral infarcts, which are not identified intraoperatively in up to 70% of HGG surgeries.^21^ Moreover, ischemic stroke secondary to HGG surgery has been reported to occur in 12.5-76% of cases, which can increase the risk of motor deficits up to 2-fold.^22^^,^^23^ The limitations of visual fluorescence analysis include the lack of sensitivity to detect fluorescence under white light and suboptimal conditions, the need to frequently switch between light sources, and the time taken to repeat the process for optimal visualization (Figure 1).

An undetected area of fluorescence almost certainly indicates an unintended residual tumor. Such residuals can be attributed to several technical factors, such as visibility obstructions caused by dead angles and the need for time-consuming switches to blue-light microscope illumination, which entails dimming the ambient lights and cleaning up blood and debris from the surgical cavity. In this context, solutions such as spectroscopic probes have been reported to improve the sensitivity for detecting fluorescence but are limited to superficial tissue layers and require invasive probes.^24^ Although the clinical benefit–risk profile of fluorescence detection beyond the visual threshold has not been established, maintaining the ability to detect fluorescence under visually suboptimal conditions and white-light illumination could significantly improve operative performance without adding to the risks of the operation.

### Intraoperative Fluorescence-Guided Aspirate Tissue Monitoring with 5-ALA

**Figure 2.**
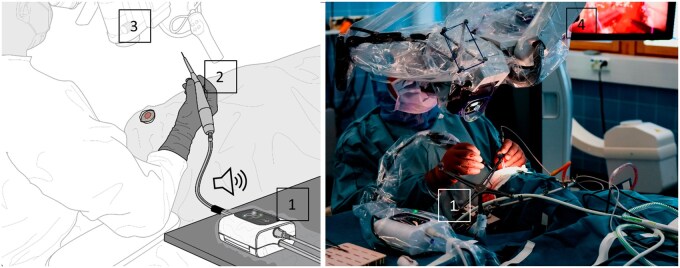
The aspirate tissue-monitoring (ATM) device (1) detects and provides auditory feedback for tumor cells on the basis of fluorescence from the surgical suction tube. The device is connected to the tubing of an active suction device, such as an ultrasonic aspirator (2). The surgeon receives auditory feedback (3 beeps) in near real-time after resection of tissues containing 5-ALA-induced PpIX fluorescence. The ATM device is a standalone device and is not affected by the illumination of the visual field of view. In contrast, tumor infiltration cannot be reliably visually detected using a surgical microscope (3) under white light (4), so blue light must be used to visualize the fluorescence. Furthermore, the reliability of visual detection of fluorescence is reduced by intensive operating room lighting, such as sunlight and ceiling lamps.

To overcome the limitations of visual analysis of PpIX fluorescence during tumor surgery, we developed and investigated a new method, aspirate tissue monitoring (ATM), that could provide surgeons with supplementary information regarding the fluorescence of resected tissues. ATM is performed with a device that can detect tumor cells based on fluorescence from the surgical suction waste in near real-time. The device is connected to an active suction device, such as an ultrasonic aspirator (Figure 2), and provides auditory feedback from the resected tissues’ pathognomonic PpIX fluorescence to the surgeon after administering 5-ALA. The device can provide near-real-time feedback on fluorescence without additional invasive steps and performs equally under any surgical microscope or theater illumination.

### Aims of the Study

We present the scientific background for the novel intraoperative ATM technique and provide data from performance analysis of the first-in-human trial experimenting with the use of the ATM device for PpIX-fluorescence detection after 5-ALA administration in patients with HGG. The performance of the ATM device was compared to that of visual analysis of fluorescence with a state-of-the-art surgical microscope. The ATM technique is demonstrated with reference to an ongoing interventional multicenter trial (ClinicalTrials.gov ref: NCT06740097) and an observational study that has enrolled and collected data from 20 patients with the first device utilizing ATM (ClinicalTrials.gov ref: NCT06736470).

Each phase of this study was launched after obtaining satisfactory results for the endpoints of the previous phase (Table 1). The preclinical investigation established an optical baseline for fluorescence detection, but the results required validation in vivo because factors such as blood may affect the results. The observational study aimed to validate the positive predictive value (PPV; identification of cases) and specificity of fluorescence detection in human tissues and investigate the clinical benefit by comparing the visual fluorescence-guided detection of fluorescence with ATM. The biopsy data from the observational study aimed to cross-validate the specificity of fluorescence detection by the ATM device for tumor detection.

**Table 1.** Description of study methods and endpoints

Study	Aims	Methods	Endpoint
Preclinical	Determine the specificity and sensitivity of the ATM device for detecting visual fluorescence	Compare visual analysis with detection using the ATM device for synthetic samples	Performance analysis of detection with the ATM device in vitro*
Observational (n = 20)	Validate the specificity and sensitivity of the ATM device for detecting visual fluorescence	Correlate visual analysis from videos with detection using the ATM device in human patients	Timeline analysis and validation of detection with the ATM device in vivo*

This manuscript presents the first-in-human evidence for the performance and safety of aspirate tissue monitoring in HGG surgery based on two investigations (preclinical and observational) and case demonstrations from an interventional trial.

* At least as good as visual.

The study explored how the device supplements contemporary surgical microscope-based visual analysis of fluorescence and adds value to fluorescence-guided HGG surgery.

## Methods

### Technical Description of Intraoperative Fluorescence-Guided ATM

The intraoperative fluorescence-guided ATM method was used to detect specific fluorescence from tissues flowing within a surgical suction tube. The ATM device (HIVEN^®^, MARGINUM Ltd, Finland) provides feedback based on tumor cell fluorescence, which is related to the PpIX concentration and has been reported in human HGG operations.^16^^,^^19^^,^^20^^,^^25^ The fluorescence on HGG can vary substantially based on cell density and microenvironment, but overall, the minimum concentration of PpIX for visual detection is between 0.6 and 1.8 μmol/L.^18^ Healthy tissues with or without 5-ALA fall below this threshold, while many other types of tumors such as lymphomas, ependymomas, and meningiomas may present with strong fluorescence.^16^ The ATM technique for 5-ALA is designed to yield supplementary information in FGS by providing near-real-time, objective, auditory feedback from tissue fluorescence indicating tumors. The device is attached to the surgical suction tube of a suction device with an active irrigation system, such as an ultrasonic aspirator. The resected tissues flow in a transparent tube that runs through the device as it analyzes the tissues through the tube and provides auditory feedback when sufficient fluorescence is detected. The investigated device has a standalone design that allows simple draping and quick configuration. The ATM device is unaffected by ambient lights and detects fluorescence under white-light illumination.

### Preclinical Evaluation of Fluorescence Detection from Surgical Suction Aspirates

Samples flowing through an ATM device can be analyzed with high performance and objectivity, as opposed to 2-dimensional or subjective visual analysis. The typical HGG tumor is strongly fluorescent, while tissues less affected by tumors show smaller concentrations of PpIX and low-intensity fluorescence. The use of 5-ALA has very little effect on the PpIX concentration in healthy tissues, which is below the visual threshold.^18^ While fluorescence indicative of active HGG tumors is, on average, strongly visible to an expert eye, tissues with less dense tumor infiltration and samples with minimal PpIX express less fluorescence. The approximate cut-off between pathognomonic fluorescence of HGGs and non-fluorescent tissues has been validated in the landmark study on 5-ALA by Stummer et al.^12^ The visual analysis of fluorescence during HGG surgery shows a high PPV for detection of PpIX fluorescence under blue light, but under white-light imaging, the detection of PpIX fluorescence is ineffective because the reddish fluorescence is mixed with light reflections. However, the NPV to rule out significant amounts of fluorescence visually under blue light is as low as 50%.^19^^,^^20^ The fluorescence of healthy tissues is negligible in contrast to the PpIX fluorescence of grade 3-4 gliomas and various other neuro-oncological entities.^16^

We investigated the feasibility of the ATM method to distinguish between tissues containing HGGs and tissues with low fluorescence by using synthetic samples mimicking brain tissues and various levels of PpIX fluorescence. Homological PpIX samples were manufactured with in vivo PpIX concentration ranges reported during resection of HGGs.^25^ The fluorescent PpIX solutions were prepared in a state-of-the-art histochemical laboratory according to the protocol described by Lehtonen et al.^18^ Non-fluorescent controls and fluorescent samples with defined concentrations of PpIX were evaluated for visual fluorescence and aspirated with a surgical suction system through the device for recording. The preclinical tests indicated that most of the sample pieces with predefined fluorescence could be detected, and non-fluorescent samples could be reliably distinguished from fluorescent samples. The investigation progressed to clinical trials after confirming that ambiguous fluorescent emissions, such as autofluorescence, would not be detected by the device during patient use. The observational clinical trial data were used to evaluate the real-life performance of the investigated ATM device.

### Study Populations

The study population consisted of 2 clinical trial cohorts: observational and interventional.

The observational trial enrolled 20 patients who had undergone surgery for suspected HGGs at a single tertiary university hospital. In the observational trial, although the ATM device data were recorded during the procedures, the device did not provide feedback to the surgeon. An international expert panel used the observational data to evaluate the fluorescence-detection performance of the device and conduct a timeline analysis between the visual and ATM detection methods. Seven neurosurgeons participated in the panel; all of them were trained in performing FGS for patients with HGGs and frequently performed glioma surgeries using 5-ALA. The experts had a cumulative neurosurgical experience of 90 years and had operated over 2400 HGGs.

An interventional study was conducted to investigate the clinical applicability of the ATM technique in a natural clinical setting. For the interventional trial, 8 participants were enrolled from three tertiary university hospitals. These participants underwent operations for suspected HGGs, which were conducted by 6 surgeons across the 3 study sites; during the surgeries, the ATM device provided feedback for fluorescence detection to the surgeons.

### Clinical Study Protocol and Data Collection

#### Perioperative surgical care and 5-ALA prescription

Surgeries for suspected HGGs, including all preoperative and postoperative procedures, were conducted according to institutional protocols and the surgeons’ preferences.

5-ALA (Gliolan™, photonamic GmbH & Co.KG, Germany) was administered orally at a dose of 1500 mg 2-5 h before the induction of anesthesia. The control cases in the observational trial were not prescribed 5-ALA or placebo. All patients in the interventional trial received 5-ALA.

#### Application of intraoperative fluorescence-guided ATM for 5-ALA

The ATM device developed for 5-ALA was attached to a suction tube of an ultrasonic aspirator to detect fluorescence. The device was toggled on and remained operational until the procedures ended. The device was calibrated with factory settings at the beginning of the experiment. After the procedure, it was detached from the suction tube for data analysis.

The device was used in 2 different operational modes depending on the study. In the observational trial, the device recorded data but did not provide feedback to surgeons. In the interventional trial, the device provided auditory feedback to surgeons when fluorescence was detected. Based on the device manufacturer’s reports, the feedback delay with optimized settings for the ultrasonic aspirator was between 0.5 and 2 s.

#### Application of visual fluorescence guidance for 5-ALA

The operations were performed by surgeons trained and qualified to use 5-ALA with visual fluorescence detection using a state-of-the-art surgical operation microscope (Kinevo™ or Pentero™; Zeiss Medical^®^). Equally maintained modern surgical operation microscopes, which had been validated for use in FGS with 5-ALA, were used to minimize potential technical bias during visual evaluation of fluorescence. Because of practical considerations, the surgeons were not blinded to the 5-ALA administration status of the patients.

#### Medical records

Patient characteristics were retrieved from medical archives. The surgical microscope and the operating room cameras recorded videos of the surgical operation, and visual fluorescence was documented in the operating surgeon’s reports.

Biopsy samples of the resected tumors were collected by the surgeon only when clinically indicated by the institutional standards. The samples were collected from representative sites while approaching the tumor as well as the margins of the resection suspected to include the tumor. Each biopsy sample was evaluated separately by a consultant neuropathologist. Pathological anatomical diagnosis (PAD) was performed for all the biopsy samples, and a combined diagnosis was based on the consensus of the neuro-oncological treatment consortium. Both preoperative and postoperative MRI images were collected from the medical imaging archive (PACS).

#### Data analysis

We compared the ATM device’s performance to the visual analysis of fluorescence with a surgical microscope. A trained clinical researcher annotated the surgical microscope videos for visual fluorescence (yes/no), and a qualified neurosurgeon validated the categories. The operation video was split into approximately 10-s sessions while the tissues were being aspirated from various surgical sites. The surgical sites were divided into sequences with only blue- or white-light illumination. The videos were presented in a randomized order to the experts, who were blinded to the device data and any earlier information for tissue fluorescence or tissue type. The experts were asked to categorize the surgical sites in the videos on the basis of fluorescence and the presence of suspected tumors in the visual field of view. The expert analysis of visual fluorescence was defined as the ground truth for fluorescence, and histological reports were used to confirm the presence of tumor growth on the respective sites, when available. The visualization of fluorescence was selected as the ground truth for performance analysis (reference) since it is less invasive than biopsies, allows numerous observation points, and has been clinically validated for safety in guiding resections.

The ATM device data were synchronized with the surgical microscope video using custom, purpose-built software (FLUOSIGHT^®^, MARGINUM). The device’s fluorescence detection (yes/no) was based on a set threshold for true-positive fluorescence detection depending on the device version.

### Setting up the Investigational Device with an Ultrasonic Aspirator during FGS with 5-ALA

The ATM device was used with two types of ultrasonic aspirators (CUSA^®^, Integra LifeSciences and Sonopet^®^, Stryker). The device worked as intended with both devices, although the settings of the ultrasonic aspirator (suction, irrigation, and power) had a variable impact on the performance.

## Results

### Patient Demographic Characteristics

Twenty participants with suspected HGGs were recruited in the observational study, and the operative videos and ATM device data were collected from the procedures. Among these 20 patients, 13 were prescribed 5-ALA, while 7 who were not prescribed 5-ALA were enrolled as controls. All participants who had been prescribed 5-ALA showed at least weak fluorescence. Strong and consistent visual fluorescence was reported in 8/11 (72%) participants with WHO grade 4 IDH-wt gliomas. Two participants with grade 4 gliomas showed only partially strong and mostly weak fluorescence based on the visual analysis. One participant showed weak fluorescence and was confirmed to have WHO grade 3 astrocytoma. None of the control participants, who were not prescribed 5-ALA, showed visual fluorescence. Six of the operated control participants had grade 2-3 oligodendrogliomas; 1 had grade 4 glioma; and 1 had focal cortical dysplasia. The patients showed no adverse events or effects related to the use of the device during the study.

### Fluorescence-Detection Performance and Safety Analysis

The expert neurosurgeons evaluated the device’s performance and safety on the basis of operation video recordings (n = 42), which included feedback from the investigational device, obtained during suspected HGG resection in 12 patients. To minimize potential context effect and bias, the evaluators were blinded to each other’s reviews, had variable degrees of expertise, and reviewed the videos in a random order.

The sensitivity and specificity of the device in comparison to visual analysis were 100% and 80%, respectively. The PPV and NPV of the device were 92% and 100%, respectively. The device detected fluorescence under white light in 11 of the resected areas, whereas experts could visually detect fluorescence under these conditions in 0 areas. Overall, 10/11 of these areas were verified by experts to show strong visual fluorescence under blue light. The remaining (1/11) area was classified as non-fluorescent, but showed fluorescence directly underneath the area that was resected in the video, which was not included in the clip due to the duration; this “false-positive” detection of fluorescence resulted from the cropping of the operation video and was not considered an actual false-positive detection. Overall, the experts were unanimous in their evaluation of visual fluorescence (38/42 evaluations agreed, agreement rate = 90.5%). The neurosurgeons agreed (average overall agreement rate = 96.7%) that the investigational device appeared safe and added value to contemporary navigation techniques.

### Fluorescence-Detection Timeline Analysis

We reviewed the operating microscope videos and the device data and recorded the lengths of time during which fluorescence-detection methods were utilized (min) and fluorescence was detected (yes/no). The amount of time that fluorescence was detected with a surgical microscope was based on the time when blue-light illumination was toggled on, and the corresponding value for the ATM device was the time when signal alterations that represent the active use of an ultrasonic aspirator were detected. The detection of fluorescence with a microscope was based on visual confirmation of fluorescence in the operational video, and that for the device was based on signal amplitudes above a set threshold. Each fluorescence-detection event was fixed to 10 s.

Five participants’ recordings were included in the timeline analysis, and the remaining were excluded because of different device versions and incompatible data. The total duration of the operating microscope videos analyzed was 552 min.

**Figure 3.**
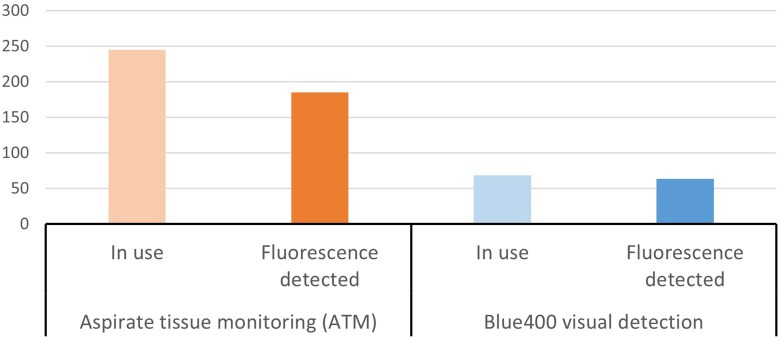
Comparison of the supplementary temporal information from fluorescence analysis in all the surgical high-grade glioma cases included in the observational analysis. The bars present minutes (min) for fluorescence screening and detection between using the ATM for fluorescence detection (left) and the microscope’s visual fluorescence detection (right). The total amount of time fluorescence was detected was 613% higher when using the ATM device in comparison with the visual analysis of fluorescence.

**Figure 4.**
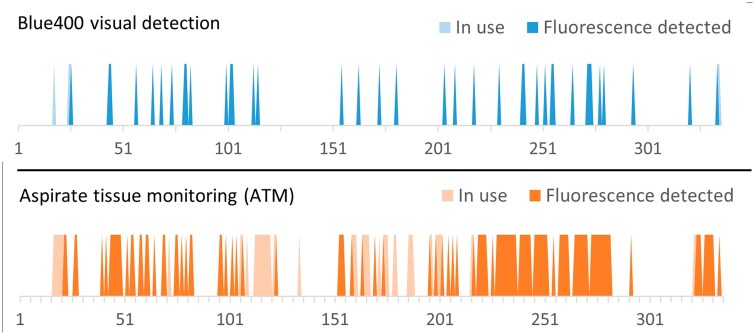
The timeline of all surgical high-grade glioma cases included in the analysis. The numbers in the x-axis represent 100 s of video. The peaks represent moments when fluorescence detection was enabled (in use) and when fluorescence was detected using visual Blue400 assessment and the ATM device. The ATM device provided intraoperative information about tissue fluorescence more often than visual analysis.

**Figure 5.**
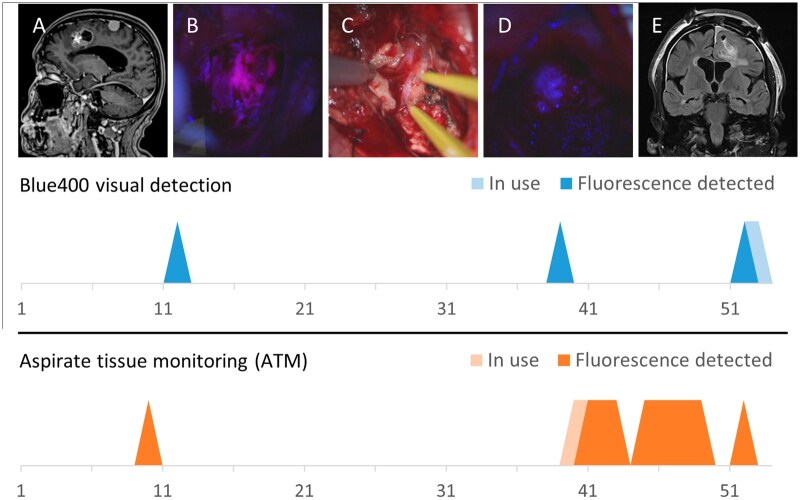
Illustration of the timelines for visual (Blue400) and ATM fluorescence detection during an HGG operation (duration 0%-100%). Preoperative MRI (A) showed a suspected HGG that was strongly fluorescent under blue light (B) but not under white light (C). The blue light was switched on, and the ultrasonic aspirator was used briefly at the beginning of the resection, but the tumor core was then removed *en bloc* with a manual dissection technique. The timeline depicts points when fluorescence was detected (solid blue and orange peaks) and when fluorescence detection was enabled but fluorescence was not detected (lighter blue or light orange). The white-light visualization revealed the callosomarginal artery (*), which was not clearly visible under the blue light. After debulking with the ultrasonic aspirator, the final field of view through the surgical microscope suggested that fluorescence was no longer visually detectable at the base of the surgical cavity (D); however, the ATM device continued to pick up signals while circling the cavity. The postoperative radiological outcome was gross total resection based on T1 contrast, but FLAIR/T2 signal abnormalities were noted (E).

The blue-light microscope was used for 68 min, accounting for 12.4% of the duration of the videos. The investigational device was active (ultrasonic aspirator in use) for 245 min (44% of the duration of the videos). The operating microscope’s blue light and the ATM device were used simultaneously for 40 min (7.2% of the total duration of the videos). Fluorescence was detected for 63 min (11.5%) under the microscopes’ blue light and for 0 min (0%) under the white light. The device detected fluorescent signal amplitudes in the resected tissues for a total of 185 min (34%), and the duration of detection under blue- and white-light illumination was equal (Figure 3). During the period of usage of the ultrasonic aspirator, the ATM device provided information regarding fluorescence 4.8 times more frequently on average ([185 min-38.33 min]/38.33 min = +382.6%) and monitored fluorescence 6.1 times longer ([245 min-40 min]/40 min = +512.5%) in comparison with blue-light assessments. A detailed timeline analysis of an illustrative HGG operation is presented in Figures 4 and 5.

### Cross-Validation of Performance in Comparison with Biopsies

In vitro diagnostic techniques such as smear and frozen-section biopsies are conventionally used for confirming the diagnosis and evaluating surgical margins between the tumor and healthy parenchyma. In the present study, targeted biopsies were used to set up diagnoses and cross-validate the presence of the tumor.

A total of 12 standard biopsy samples were collected from areas with suspected tumors from the borders of the surgical cavity. The biopsy samples were obtained from 5 patients with histologically confirmed WHO grade 4 IDH-wt gliomas. The device’s positive-feedback rate for predicting tumors on biopsy sites was 100%, and it showed no false-positive findings. For clarity, while the device detects the fluorescence generated by the 5-ALA metabolite PpIX, it does not directly detect tumor cells.

The results from performance and timeline analysis and expert feedback have been summarized in Table 2.

**Table 2.** Comparison of performance between the visual and the investigated ATM for the detection of 5-ALA fluorescence during HGG surgeries

Performance, *n* (%)	White light		Blue400 light		
	Microscope	ATM	Microscope	ATM	
Fluorescence, yes	0 (0 %)	11 (69 %)	11 (69 %)	12 (75 %)	PPV 92 %
Fluorescence, no	16 (100 %)	5 (31 %)	5 (31 %)	4 (25 %)	NPV 100 %
			Spesificity 100 %	Sensitivity 83 %	

Validation, *n* (%)	HGG in PAD	
Biopsies*, yes	6 (100 %)	PPV 100 %

**Timeline**, min (+ %)	Microscope	ATM
Detection mode inactive	512	307 (60%)
Detection mode active	40	245 (513%)
Fluorescence detected	38	185 (383%)

The table depicts the summary of analyses evaluating the ATM performance for the detection of visually confirmed fluorescence in comparison to the state-of-the-art visual analysis and histopathology. The timeline analysis describes the total duration for screening (detection mode active) and detecting (fluorescence detected) between the methods.

Legend: PPV = positive predictive value, NPV = negative predictive value, HGG = high-grade glioma.

* From a visually fluorescent target.

## Discussion

### Clinical Performance of Intraoperative Fluorescence-Guided ATM during HGG Surgery

In the present set of preclinical and clinical studies, the performance of the novel intraoperative fluorescence-guided ATM device was compared with visual fluorescence-guided detection. Our results indicated that the device was at least as reliable as visual analysis for obtaining information regarding tissue fluorescence and substantially enhances sensitivity to detect clinically relevant tumor-related fluorescence under white-light illumination. The ATM technique allowed constant monitoring of fluorescence irrespective of the illumination color as opposed to the active but intermittent detection using the surgical microscope’s blue-light filters.

Analysis based on 5-ALA-induced PpIX fluorescence continues to be the most precise contemporary method for near-real-time detection of active tumor cells. Our findings confirmed several expected clinical benefits of using the ATM method during HGG surgery, with the device essentially providing supplementary feedback that supported decision-making during the resection. The investigational ATM device provided information about detected fluorescence nearly 5 times more frequently and 6 times longer during the procedures. All visually detected fluorescent areas were histopathologically verified to contain HGGs and compared to feedback from the ATM device; this resulted in a highly reliable PPV for visual fluorescence. Meanwhile, the risk of false-positive interpretation errors appeared to be very low; the infrequent false-positive signals are likely attributable to stuck particles and delayed flow through the suction tube. Blood in the tube walls frequently limited the detection ability of the ATM device; however, this could be minimized by increased flushing. Moreover, while the device feedback was occasionally difficult to hear in the operating room, this could be overcome by moving the device closer to the operator. The learning curve while adopting the device was not fully addressed within this study because of the small number of operators and cases in the interventional study, but it remains an interesting field for future research.

### Role of Intraoperative Near-Real-Time Tissue Detection in Clinical Outcomes

Near-real-time tissue detection with neuronavigation, ultrasound, and fluorescence guidance has been widely adopted to increase the EOR in HGG surgery. Although tools such as ultrasonic aspirators have made resection along the tumor margins easier, their role in increasing the EOR is less clear. However, these active soft tissue suction systems have undergone further advancements for tissue-sensitive modules such as continuous dynamic mapping, as described originally by Raabe et al.^26^ Similarly, the investigated intraoperative fluorescence-guided ATM method allows suction systems to provide information on tissue features but also provides supplementary information on fluorescence during HGG surgery, which may ultimately play a role in the surgical treatment strategy.

Our expert panel and observational data indicate that using the ATM method can allow precise and unobtrusive near-real-time detection of fluorescence with a performance comparable to that of visual fluorescence guidance with 5-ALA. Based on expert opinions regarding the investigational device’s performance, the ATM method appears to be a safe method that enables maximizing the EOR of gliomas. The ATM method’s ability to detect fluorescence in deep surgical cavities with a limited field of view and during white-light microscope illumination represents a major benefit in comparison to visualization with blue light alone.

### Implications of Using the ATM Method on Surgical Strategies for HGGs

The ATM method was designed to provide supplementary information regarding tissue fluorescence. For most grade 3-4 gliomas, the PpIX fluorescence extends beyond the contrast-enhancing region on MRI as well as 18F-fluoroethyl-tyrosine positron emission tomography ([^18^F] FET-PET) imaging findings.^27^^,^^28^ Grade 4 gliomas nearly always show strong 5-ALA-induced fluorescence, although the tumor can diffusely extend beyond the radiological boundaries and FLAIR/T2 hyperintense zones.^29^ FLAIR signal abnormalities and T2 hyperintensities have often been associated with PpIX fluorescence on low-grade gliomas as well.^30^ Thus, more information regarding tissue fluorescence during resections can facilitate better selection of resection strategies targeting the diffuse tumor microenvironment, and fluorescence can help identify the margin for supramarginal resections. Meanwhile, achieving a clean margin based on feedback from the ATM method may suggest that the resection was incomplete, which could guide further treatment decisions. In our expert panel review, all experts agreed that the ATM provides supplementary information on fluorescence, and most experts believed that its use could lead to improved care.

### Limitations

The number of biopsies in our studies was limited, but the findings showed consistent associations between the detected fluorescence and the tumors, with no false-positive correlations. Although the ATM device is characterized by a high NPV owing to its design, confirming this in a clinical setting was challenging. The ultrasonic aspirator’s settings had a notable effect on the performance of the device, although the frequency of fluorescence detection improved with optimized settings, and the auditory feedback was near real-time. More studies are needed to define the optimal system-specific settings and applications of the ATM technique. Although the ATM technique can support resection strategies targeting FLAIR/T2 signal abnormalities, further clinical studies are warranted to clarify and substantiate the overall clinical benefits.

## Conclusions

This is the first clinical study investigating the clinical benefits of the ATM device for detection of 5-ALA-induced fluorescence in FGS for HGGs. The findings and expert opinions indicated that the ATM device yielded beneficial supplementary information pertaining to tumor-related fluorescence during HGG surgery. The ATM device appeared to simplify the surgical workflow during FGS. The practical benefits of using the ATM device included the ability to passively monitor fluorescent features, enabling the resection of HGG under natural white light. The use of the ATM device is expected to enable more aggressive tumor-resection strategies and support more informed decision-making when choosing between the GTR and supramarginal resection strategies.

## Data Availability

The research data is not publicly available for research use. The preclinical and clinical data used in this study are restricted to regulatory inspections alone.

## Clinical Trials

The observational clinical study presented in this manuscript is registered to ClinicalTrials.gov (NCT06736470). Further studies will be sought to investigate the incremental practical and clinical benefits from using the ATM method and device in conjunction with other surgical adjunct methods. Our research group has received approval for a clinical controlled trial exploring long-term clinical outcomes from using ATM with 5-ALA in conjunction with other adjunct tissue mapping techniques (ClinicalTrials.gov ID: 5252675). This interventional international multicenter study collects unsupervised user experiences; it follows up on EOR, PFS, OS, and clinical performance in addition to intraoperative performance measures such as blood loss and duration of the procedure.
